# Supplementary material for: Engineering advanced logic and distributed computing in human CAR immune cells
Source: Nat Commun. 2021 Feb 4;12:792. doi: 10.1038/s41467-021-21078-7 (PMC7862674; doi:10.1038/s41467-021-21078-7)
Supplement: Supplementary file 1 — Supplementary Information [file 41467_2021_21078_MOESM1_ESM.pdf]

Supplementary Information for:

Engineering Advanced Logic and Distributed Computing in Human CAR Immune Cells

Jang Hwan Cho[1,2], Atsushi Okuma[1,2], Katri Sofjan[1,2], Seunghee Lee[1,2], James J. Collins[3,4,5,6,7,8], and Wilson W. Wong[1,2*]

[1]Department of Biomedical Engineering, Boston University, Boston, MA 02215, USA.
[2]Biological Design Center, Boston University, Boston, MA 02215, USA.
[3]Synthetic Biology Center, MIT, Cambridge, MA 02139, USA
[4]Institute for Medical Engineering and Science, MIT, Cambridge, MA 02139, USA
[5]Department of Biological Engineering, MIT, Cambridge, MA 02139, USA
[6]Harvard-MIT Program in Health Sciences and Technology, Cambridge, MA 02139, USA
[7]Broad Institute of MIT and Harvard, 415 Main Street, Cambridge, MA 02142, USA
[8]Wyss Institute for Biologically Inspired Engineering, Harvard University, 3 Blackfan Circle, Boston, MA 02115, USA
These authors contributed equally: Jang Hwan Cho, Atsushi Okuma
*Correspondence: wilwong@bu.edu.

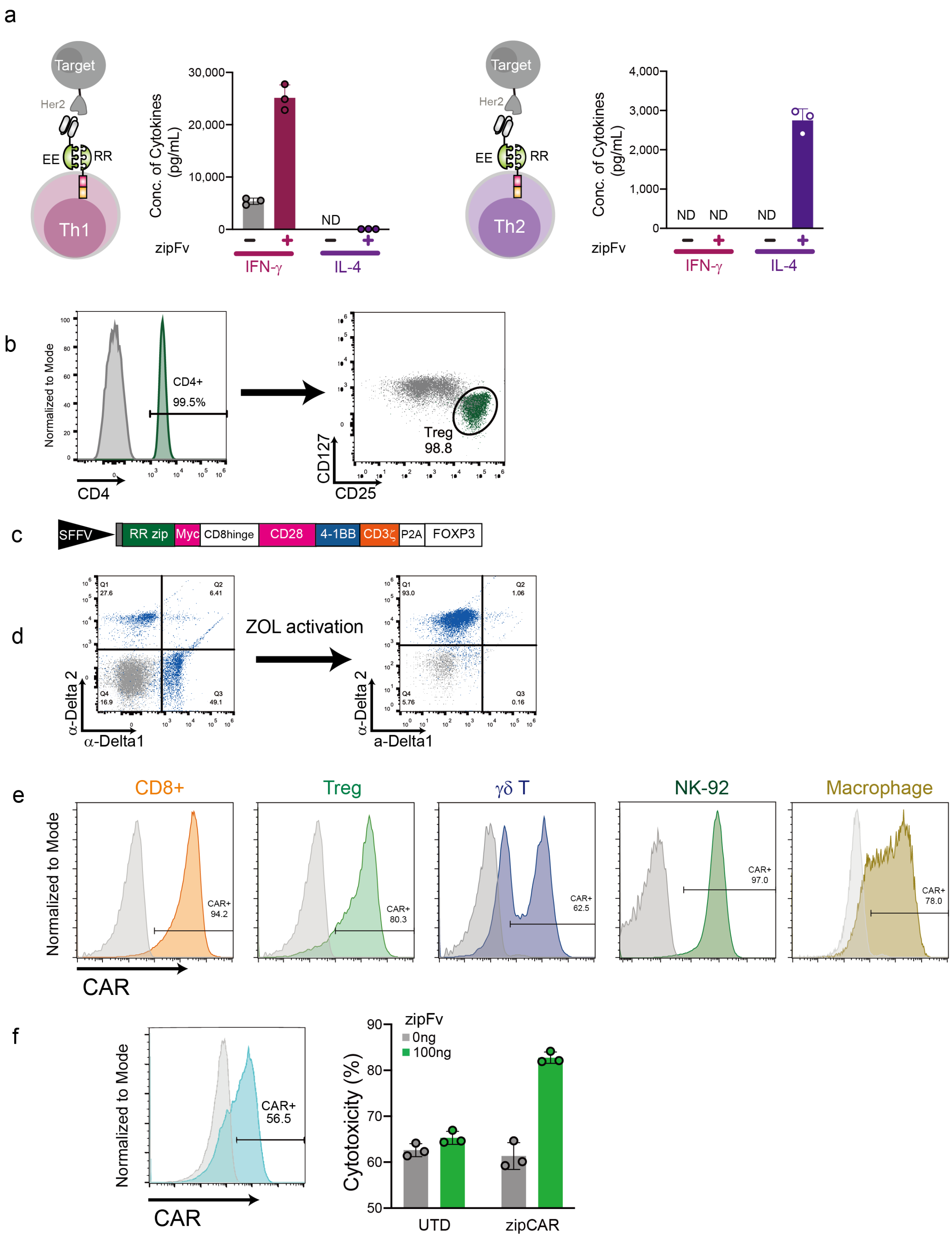

Supplementary Fig. 1| Different immune cell types expressing SUPRA CAR Related to Fig. 1

a. (Left) IFN-γ and IL-4 cytokine secretion from RR zipCAR expressing *in vitro* differentiated Th1 cells (Right) IFN-γ and IL-4 cytokine level from RR zipCAR expressing *in vitro* differentiated Th2 cells. Nalm6 cells expressing Her2 were co-cultured with RR zipCAR expressing Th1 and Th2 cells with and without α-Her2-EE zipFv (n=3, data are represented as mean + SD).

b. Verification of the isolated CD4+CD25^{high}CD127^{low} Treg cells. Once Treg cells have been isolated using “EasySep™ Human CD4+CD127^{low}CD25+ Regulatory T Cell Isolation Kit” (STEMCELL #18063), Treg surface markers (CD4, CD127, and CD25) have been verified using flow cytometry (Isolated Treg population is colored in green)(representative of three biological replicates).

c. Schematic of zipCAR construct used for Treg cell experiments. FOXP3 transcription factor was coexpressed with zipCAR using P2A ribosomal skipping sequence to enhance Treg cell stability.

d. Verification of $\gamma\delta$ T cell surface marker after ZOL activation. Once $\gamma\delta$ T cells were isolated using EasySep™ Human Gamma/Delta T Cell Isolation Kit (STEMCELL Car#19255), isolated cells were activated using zoledronic acid. $\gamma\delta$ T specific surface markers (both Vδ 1 and Vδ 2) were used to verify the expression of beta TCR from isolated $\gamma\delta$ T cells (representative of three biological replicates).

e. CAR expression in different immune cell types was verified by the expression of the mCherry that was fused to zipCAR (representative of three biological replicates, gray represents untransduced or nonfluorescence cells).

f. (Left)CAR expression of SUPRA CAR in human primary NK cells.   (n=3, data are represented as mean + SD, gray represents untransduced or unfluorescent cells) (Right)Target cell killing of SUPRA CAR in human primary NK cells (n=3, data are represented as mean + SD).

a

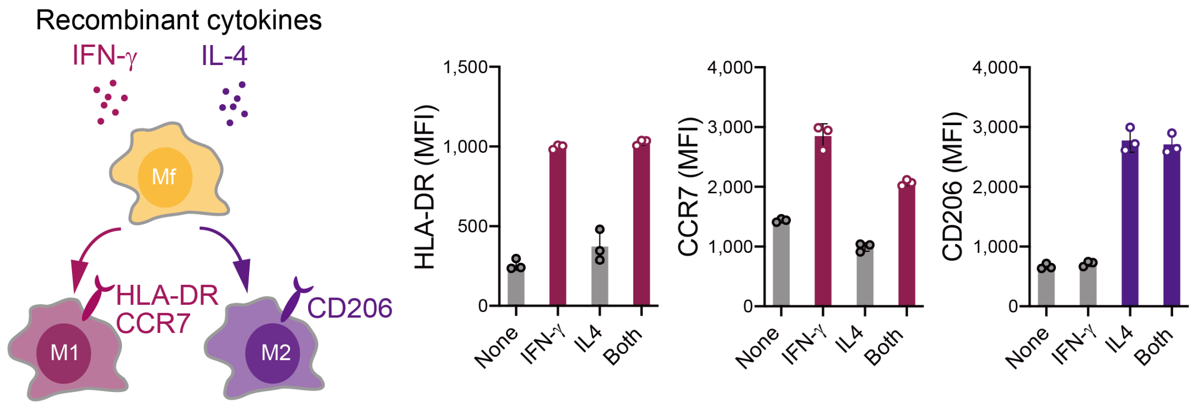

b

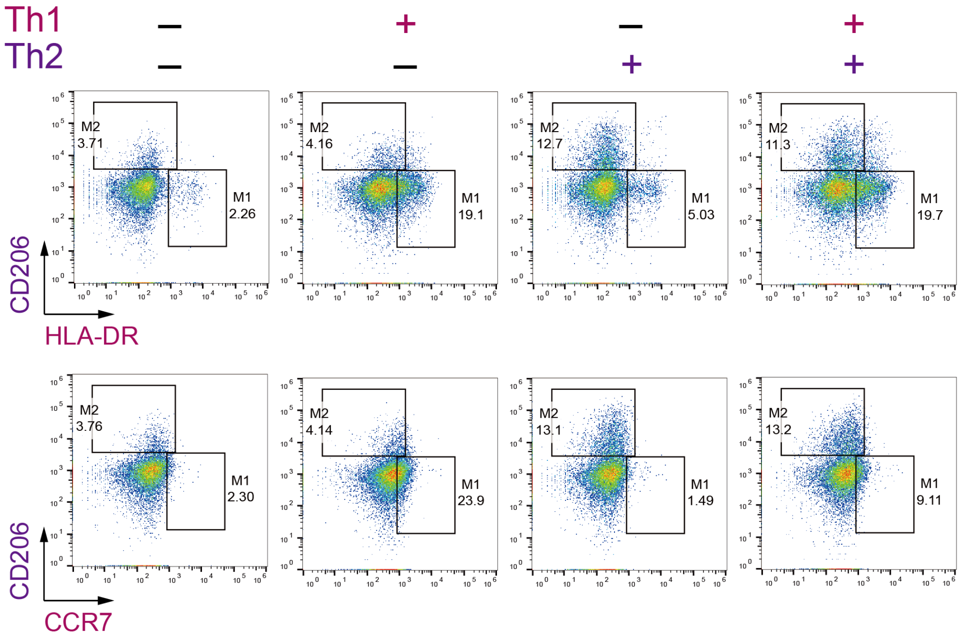

Supplementary Fig. 2| Effect of recombinant cytokines and engineered Th1/Th2 cells on macrophage polarization, Related to Fig. 2

a. Effect of recombinant IFN-γ (10 ng/mL) or IL-4 (10 ng/mL) on *in vitro* macrophage polarization. M1 and M2 macrophage surface markers are measured 24 hr after adding cytokines (n=3, data are represented as mean + SD). b. Flow cytometry plot shown in Fig. 2c.

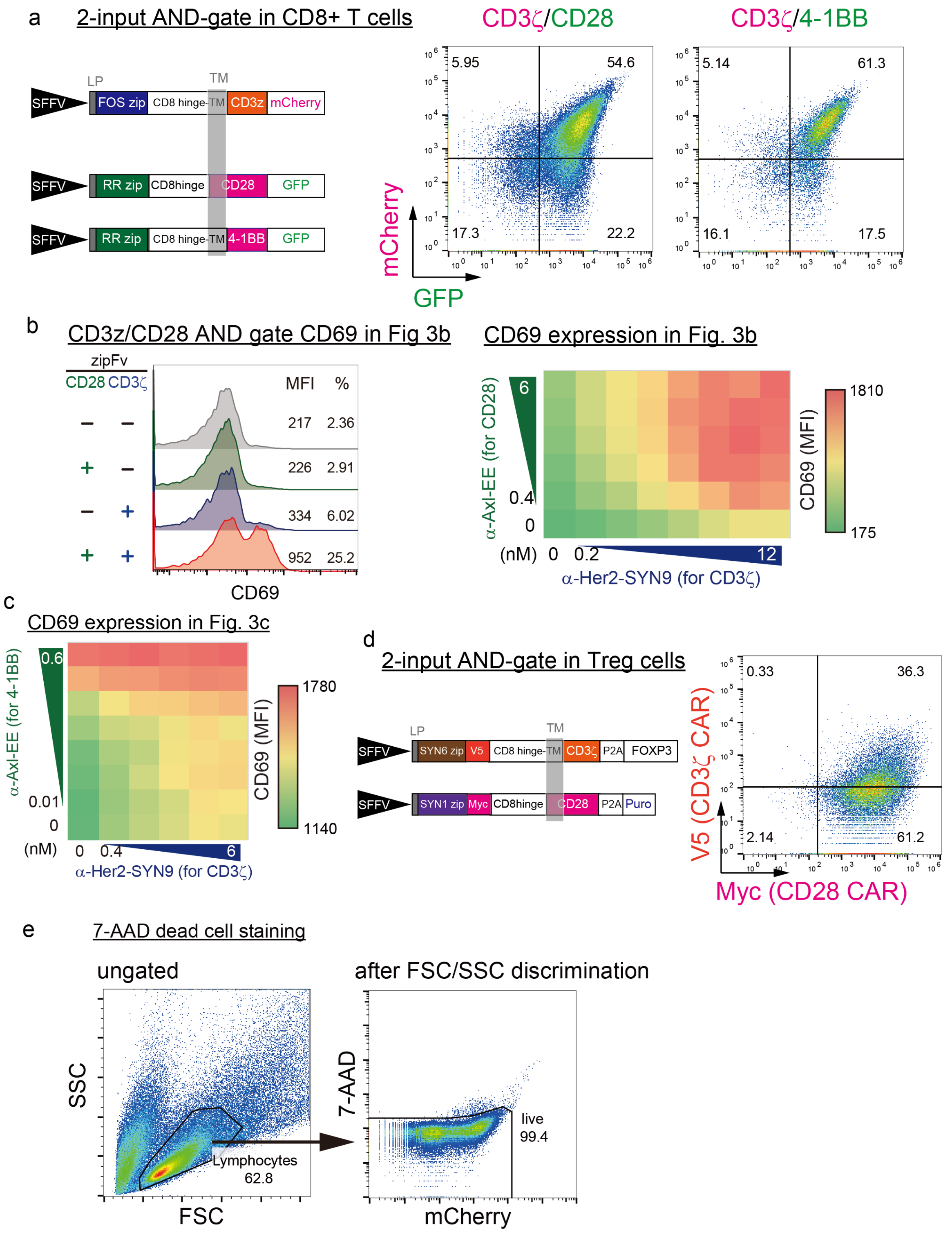

Supplementary Fig. 3| Schematics of SUPRA CARs used for 2-input AND gates, Related to Fig. 3 a. (Left) Schematics of 2 input AND gate constructs. FOS zipCAR is used to control CD3ζ domain and RR zipCAR is used to control CD28 or 4-1BB co-stimulatory domains. Both

zipCARs are fused to GFP or mCherry for visualization (Right) FACS diagram that shows the expression of two different receptors in a single cell (representative of three biological replicates). b. CD69 expression on 2-input CD3z/CD28 AND gate T cells shown in Fig. 3b. c. CD69 expression on 2-input CD3z/4-BB AND gate T cells shown in Fig. 3c. d. (Left) Schematics of 2 input AND gate construct designs. SYN5 zipCAR is used to control CD3ζ domain and SYN1 zipCAR is used to control CD28 co-stimulatory domain. FOXP3 transcription factor and puromycin resistance gene are co-expressed with zipCARs using P2A ribosomal skipping sequence. After transduction into primary T cells, cells were treated with 2 μg/mL puromycin to select for positive cells (Right) FACS diagram that shows the expression of two different receptors in a single cell (representative of three biological replicates). e. Dead cell discrimination to count live cells. The indicating gate on FSC/SSC plots can discriminate dead cells, and more than 99% of cells are 7-AAD negative.

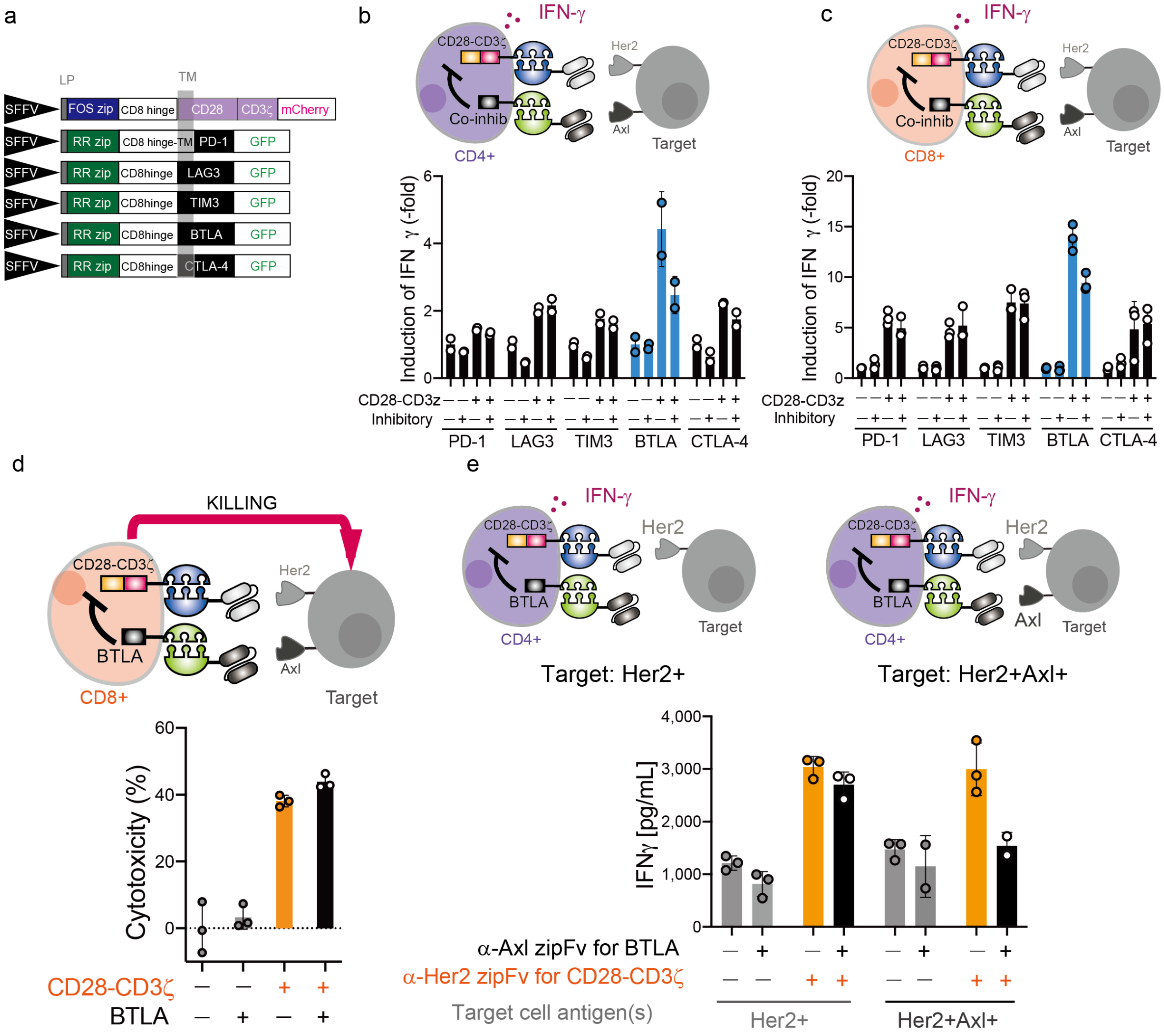

Supplementary Fig. 4| Co-Inhibitory domain screening in T cells, Related to Fig. 4

a. Schematics of co-inhibitory domain constructs. FOS zipCAR controls CD28-CD3ζ domain and RR zipCAR regulates different co-inhibitory domains including PD-1, LAG3, TIM3, BTLA, and CTLA-4.

b-c. Diagram of co-inhibitory screening in CD4+ T cells (B) and CD8+ T cells (C). CD4+ and CD8+ T cells are engineered to express FOS-CD28-CD3ζ zipCAR (binds to α-Her2-SYN9 zipFv) and RR zipCAR (binds to α-Axl-EE zipFv) with different co-inhibitory domains. IFN-γ cytokine level is measured after adding different combinations of zipFvs (4b, n=2; 4c, n=3; data are represented as mean + SD).

d. Cytotoxicity of CD8+ T cells transduced with FOS-CD3ζ and RR zipCAR with BTLA co-inhibitory domain. Live NALM-6 target cells were counted 24 hours after adding 1.2 nM α-Her2-SYN9 zipFv and/or 12 nM α-Axl-EE zipFv. (n=3, data are represented as mean + SD).

e. IFN-γ production from CD4+ T cells transduced with FOS-CD28-CD3ζ and RR-BTLA against Her2+ only (Left) and Axl+/Her2+ (Right) Nalm6 target cells. Supernatants are collected 24

hours after incubation with 2.4 nM α-Her2-SYN9 zipFv and/or 12 nM α-Axl-EE zipFv (n=2 in the conditions targeting Her2+Axl+ with α-Axl-EE zipFv, n=3 in the other conditions; data are represented as the mean + SD)

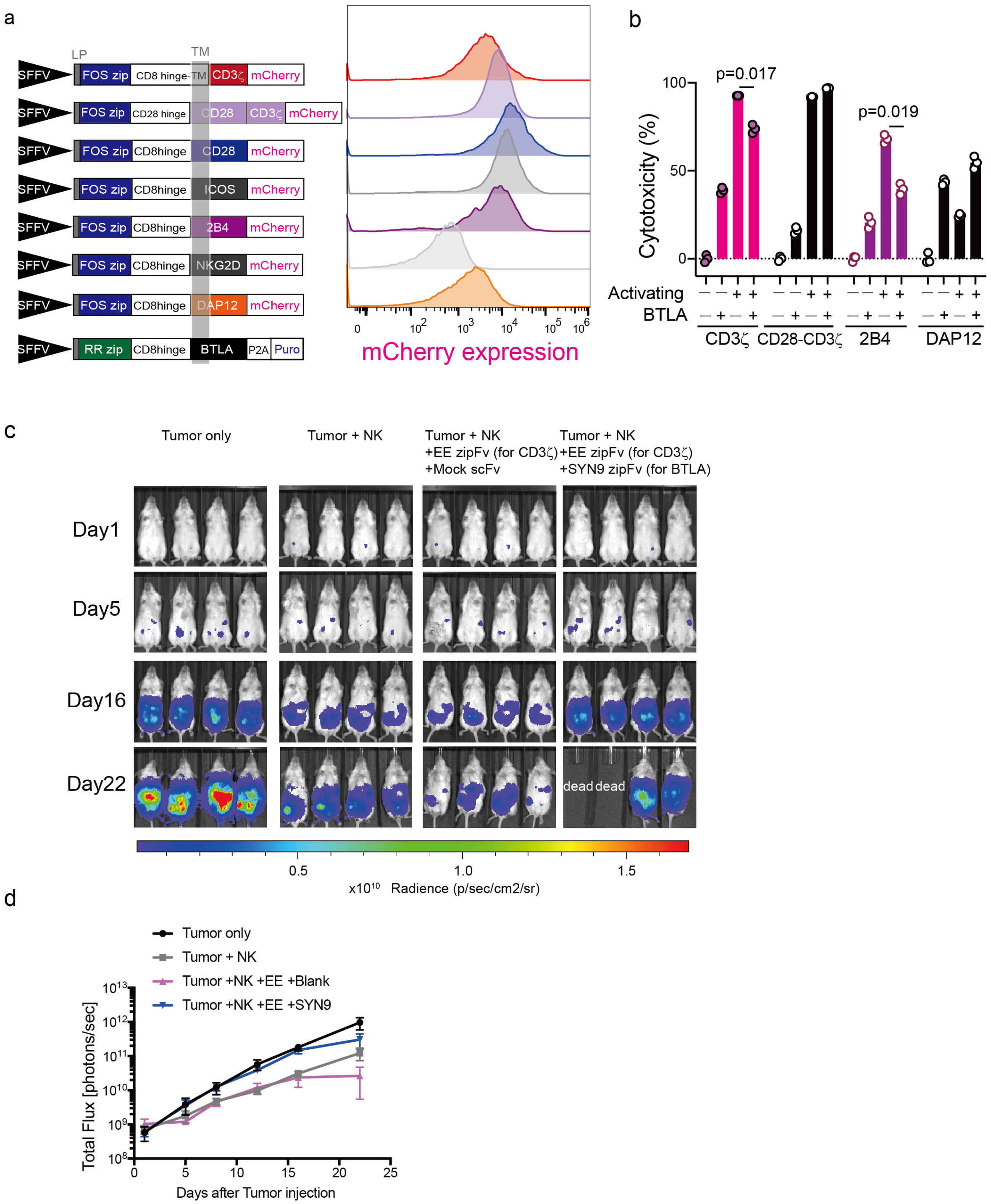

Supplementary Fig. 5| Co-stimulatory and co-inhibitory domain screening in NK cells, Related to Fig. 4

a. (Left) Schematics of activating domains and co-inhibitory domain constructs used in NK cells. FOS zipCAR controls different activating domains including CD3ζ, CD28-CD3ζ, CD28, ICOS, 2B4, NKG2D, and DAP12. RR zipCAR regulates BTLA co-inhibitory domain (Right) zipCAR expression level measured by flow cytometry (representative of three biological replicates).

b. Effect of BTLA activation on different activating domains in NK cell. NK cells were transduced with FOS CAR having CD3ζ, CD28-CD3ζ, 2B4, or DAP12 activating domain and RR zipCAR having BTLA inhibitory domain. Cytotoxicity was measured 24 hr after adding 1.2 nM α-Axl SYN9 zipFv and/or 6 nM α-Her2 EE zipFv (n=3; data are represented as mean + SD; the statistical significance was determined by two-tailed student's T-test).

c. Representative IVIS images of groups shown in Fig. 4g (n=4).

d. Quantified tumor burden from the luciferase activity of each mouse shown in Supplementary Fig. 5c (n=4; data are represented as mean ± SD).

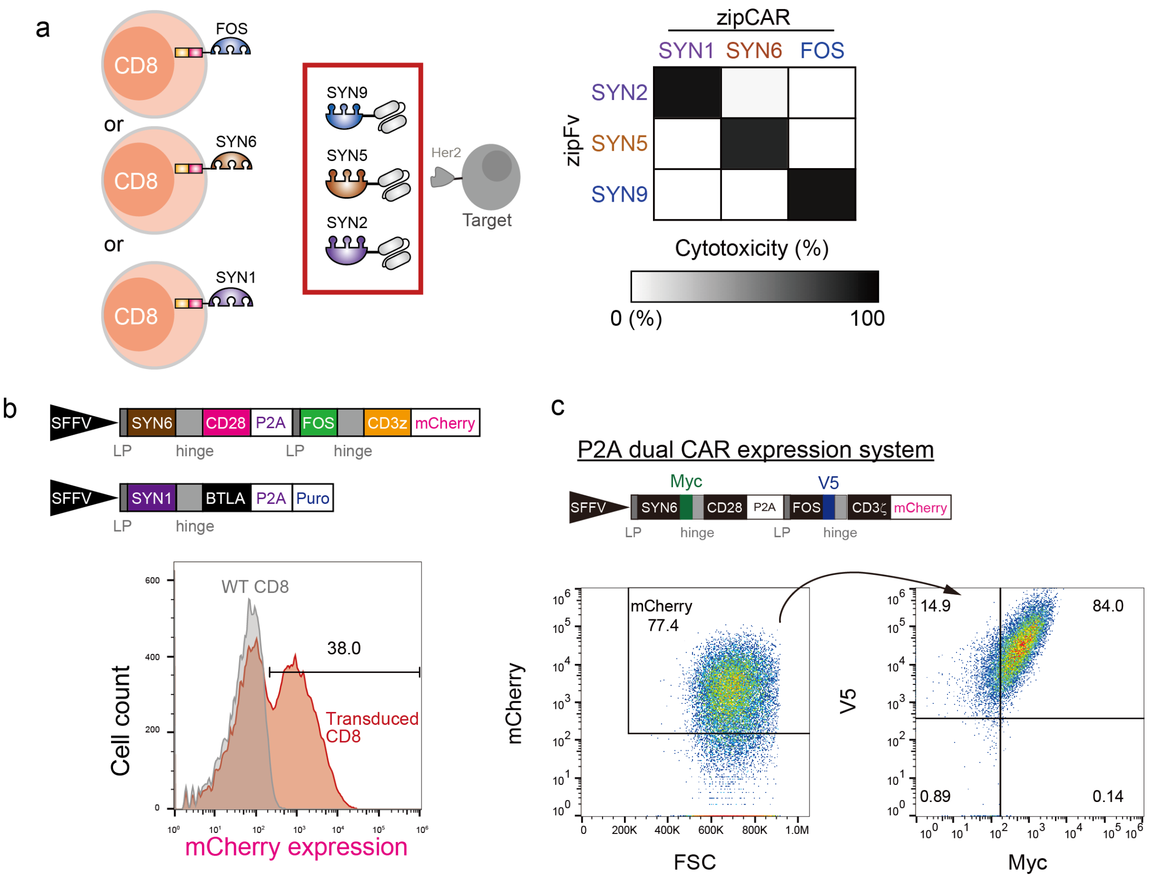

Supplementary Fig. 6| Orthogonal SUPRA pairs in primary human T cells and schematics construct designs used for 3 input logic circuit, Related to Fig. 5

a. Functional orthogonal SUPRA pairs in CD8+ T cells. CD8+ T cells were transduced with FOS zipCAR, SYN2 zipCAR, or SYN6 zipCAR. Each type of SUPRA CAR T cells was co-cultured with Her2+ Jurkat target cells. Live target cells were measured 24 hr after adding α-Her2 SYN1, SYN6, and SYN9 zipFv, respectively (n=3, data are represented as mean).

b. (Left) Schematics of three different zipCAR constructs used for 3 input logic circuits (Right) zipCAR expression level measured by flow cytometry after puromycin selection.

c. P2A dual CAR expression system demonstrates high percentage of mCherry positive cells are both myc-tag and V5-tag positive.

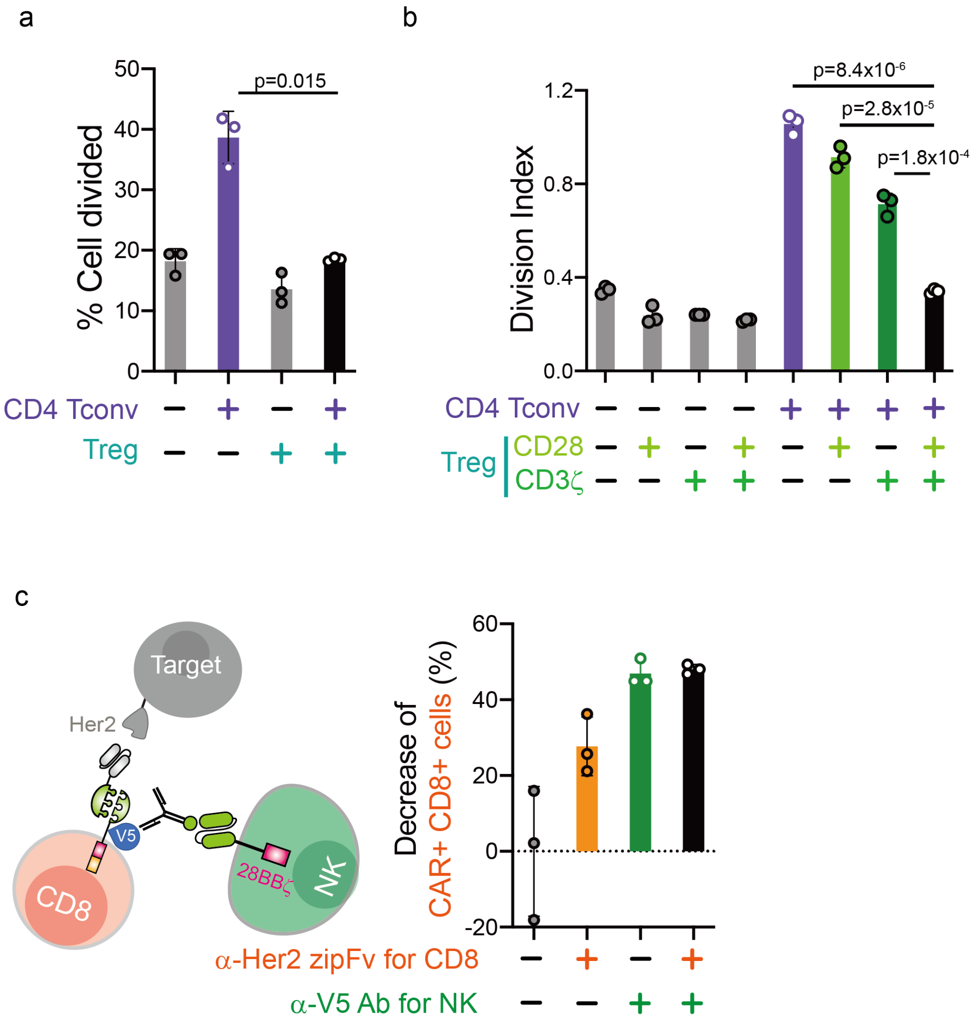

Supplementary Fig. 7| Different logic gates circuits using multiple immune cell types, Related to Fig. 6

a. Quantified suppression of CD4+ conventional T cell growth by SUPRA CAR-expressing Treg cells in Fig. 6b (n=3; data are represented as mean + SD; the statistical significance was determined by two-tailed student's T-test; **, $p=1.3X10^{-3}$).

b. Quantified suppression of CD4+ conventional T cell growth by SUPRA CAR-expressing Treg cells in Fig. 6d (n=3; data are represented as mean + SD; the statistical significance was determined by two-tailed student's T-test; ***, $p<0.001$).

c. Decrease of SUPRA CAR+ CD8+ cells in Fig. 6e-g. The number of SUPRA CAR+ CD8+ cells were counted by flow cytometry at the same timing as Fig. 6g (n=3; data are represented as mean + SD).
