# Supplementary material for: Engineering advanced logic and distributed computing in human CAR immune cells
Source: Nat Commun. 2021 Feb 4;12:792. doi: 10.1038/s41467-021-21078-7 (PMC7862674; doi:10.1038/s41467-021-21078-7)
Supplement: Supplementary file 2 — Reporting Summary [file 41467_2021_21078_MOESM2_ESM.pdf]

nature research

Corresponding author(s): Wilson Wong

Last updated by author(s): Dec 27, 2020

Reporting Summary

Nature Research wishes to improve the reproducibility of the work that we publish. This form provides structure for consistency and transparency in reporting. For further information on Nature Research policies, see Authors & Referees and the Editorial Policy Checklist .

Statistics

For all statistical analyses, confirm that the following items are present in the figure legend, table legend, main text, or Methods section.

n/a	Confirmed	
☐	☒	The exact sample size (*n*) for each experimental group/condition, given as a discrete number and unit of measurement
☐	☒	A statement on whether measurements were taken from distinct samples or whether the same sample was measured repeatedly
☐	☒	The statistical test(s) used AND whether they are one- or two-sided<br>*Only common tests should be described solely by name; describe more complex techniques in the Methods section.*
☒	☐	A description of all covariates tested
☒	☐	A description of any assumptions or corrections, such as tests of normality and adjustment for multiple comparisons
☐	☒	A full description of the statistical parameters including central tendency (e.g. means) or other basic estimates (e.g. regression coefficient) AND variation (e.g. standard deviation) or associated estimates of uncertainty (e.g. confidence intervals)
☒	☐	For null hypothesis testing, the test statistic (e.g. *F*, *t*, *r*) with confidence intervals, effect sizes, degrees of freedom and *P* value noted<br>*Give P values as exact values whenever suitable.*
☒	☐	For Bayesian analysis, information on the choice of priors and Markov chain Monte Carlo settings
☒	☐	For hierarchical and complex designs, identification of the appropriate level for tests and full reporting of outcomes
☒	☐	Estimates of effect sizes (e.g. Cohen's *d*, Pearson's *r*), indicating how they were calculated

Our web collection on statistics for biologists contains articles on many of the points above.

Software and code

Policy information about availability of computer code

Data collection	Attune Nxt Software 2
Data analysis	FlowJo 10 (TreeStar); Microsoft Excel 2011; Living Images (Perkin Elmer); Graphpad Prism

For manuscripts utilizing custom algorithms or software that are central to the research but not yet described in published literature, software must be made available to editors/reviewers. We strongly encourage code deposition in a community repository (e.g. GitHub). See the Nature Research guidelines for submitting code & software for further information.

Data

Policy information about availability of data

All manuscripts must include a data availability statement. This statement should provide the following information, where applicable:

- Accession codes, unique identifiers, or web links for publicly available datasets
- A list of figures that have associated raw data
- A description of any restrictions on data availability

Data are available from the corresponding authors upon reasonable request.

Field-specific reporting

Please select the one below that is the best fit for your research. If you are not sure, read the appropriate sections before making your selection.

☒ Life sciences ☐ Behavioural & social sciences ☐ Ecological, evolutionary & environmental sciences

For a reference copy of the document with all sections, see nature.com/documents/nr-reporting-summary-flat.pdf

Life sciences study design

All studies must disclose on these points even when the disclosure is negative.

Sample size	We chose to do at least two replicates for each experiments. Each sample size was specified in the figure legend. Sample size was calculated based on the previous studies (ref.10)
Data exclusions	No data was excluded.
Replication	Each experiment was repeated at least two times with more than 2 technical replicates except in vivo experiment and gdT cell experiment.
Randomization	No randomization was used. All samples used were from the same homogeneous mix of cells and therefore eliminated possible covariates between different experimental groups.
Blinding	No blinding was used in experiments used cell lines that should not be affected by the experimenter. Tumor measurements were tried to be done by blind test initially, but after some mice died in the specific group, the blinding has not been perfect anymore (fig..4g).

Reporting for specific materials, systems and methods

We require information from authors about some types of materials, experimental systems and methods used in many studies. Here, indicate whether each material, system or method listed is relevant to your study. If you are not sure if a list item applies to your research, read the appropriate section before selecting a response.

Materials & experimental systems

n/a	Involved in the study	
☐	☒	Antibodies
☐	☒	Eukaryotic cell lines
☒	☐	Palaeontology
☐	☒	Animals and other organisms
☐	☒	Human research participants
☒	☐	Clinical data

Methods

n/a	Involved in the study	
☒	☐	ChIP-seq
☐	☒	Flow cytometry
☒	☐	MRI-based neuroimaging

Antibodies

Antibodies used	anti-Myc (Abcam #ab62928), anti-CD3-Alexa Fluor 647 antibody (1:100 dilution, Biolegend, #300322), anti-CD56-APC-Cy7 (1:100 dilution, Biolegend, #362511), anti-CD3 (Thermo Fisher, clone OKT-3, #14-0037-82), APC-conjugated anti-CD69 antibody (1:200 dilution, BioLegend #310910), FITC-conjugated anti-CTLA4 antibody (1:50 dilution, Thermo Fisher Scientific #11-1529-42)<br>APC anti-human CD44 antibody (1:100 dilution, Biolegend #338806), APC anti-HLA-DR (1:100 dilution, BioLegend #307609), APC anti-CCR7 (1:100 dilution, BioLegend #353214), PE anti-CD206 (1:100 dilution, BioLegend #321105), FITC-conjugated anti-V5 tag antibody (Thermo Fisher Scientific #R963-25), BV421 Mouse Anti-Human CD8 (1:200 dilution, BD, clone RPA-T8),<br>Anti-Human CD19 PE-Cy7 (1:200 dilution, Tonbo Bioscience, clone HIB19), FITC Mouse Anti-Human CD4 (1:200 dilution, BD, clone M-T477), Pacific Blue™ Mouse Anti-Human CD8 (1:200 dilution, BD, clone RPA-T8), APC anti-human TCR Vδ2 Antibody (1:100 dilution, Biolegend, #331418), Alexa Fluor 647 anti-V5 Tag (1:100 dilution, R&D systems), Alexa Fluor 405 Anti-c-Myc (1:100 dilution, Novus bio), Alexa Fluor 647 anti-Myc Tag (1:100 dilution, Cell Signaling Technology), BV510 Mouse Anti-Human CD25 Clone 2A3 (1:100 dilution, BD Bioscience, #740198), BV711 Mouse Anti-Human CD25 Clone 2A3 (1:100 dilution, BD Bioscience, #563159), Alexa Fluor® 647 Mouse anti-Human FoxP3 Clone 259D/C7 (1:100 dilution, BD Bioscience, #560045), Brilliant Violet 421™ anti-human TCR α/β Antibody (1:100 dilution, Biolegend #306722)
Validation	Validation statements, as well as references from the literature, can be found on the manufacturers' websites.

Eukaryotic cell lines

Policy information about cell lines

Cell line source(s)	Freestyle 293-F cells (Thermo Fisher Scientific #R79007), HEK293FT cells (Thermo Fisher Scientific #R70007), K562 myelogenous leukemia cells (ATCC #CCL-243), Jurkat T cells, NALM-6 B cell precursor leukemia (ATCC #CRL-3273), THP-1 (kindly gifted from Siggers lab, Boston University)
Authentication	No authentication was made. Cells were purchased directly from source
Mycoplasma contamination	We did not test for mycoplasma contamination.

Commonly misidentified lines (See ICLAC register) | None of misidentified lines on the list were used in this study.

Animals and other organisms

Policy information about studies involving animals; ARRIVE guidelines recommended for reporting animal research

Laboratory animals	Female NSG mice, 4-6 weeks of age, were purchased from Jackson Laboratories
Wild animals	No wild animal were used in this study.
Field-collected samples	No field collected samples were used in this study.
Ethics oversight	All protocols of animal experiments were approved by the Institutional Animal Care and Use Committee at BUMC.

Note that full information on the approval of the study protocol must also be provided in the manuscript.

Human research participants

Policy information about studies involving human research participants

Population characteristics	*Describe the covariate-relevant population characteristics of the human research participants (e.g. age, gender, genotypic information, past and current diagnosis and treatment categories). If you filled out the behavioural & social sciences study design questions and have nothing to add here, write "See above."*
Recruitment	*Describe how participants were recruited. Outline any potential self-selection bias or other biases that may be present and how these are likely to impact results.*
Ethics oversight	*Identify the organization(s) that approved the study protocol.*

Note that full information on the approval of the study protocol must also be provided in the manuscript.

Flow Cytometry

Plots

Confirm that:

- [x] The axis labels state the marker and fluorochrome used (e.g. CD4-FITC).
- [x] The axis scales are clearly visible. Include numbers along axes only for bottom left plot of group (a 'group' is an analysis of identical markers).
- [x] All plots are contour plots with outliers or pseudocolor plots.
- [x] A numerical value for number of cells or percentage (with statistics) is provided.

Methodology

Sample preparation	Isolated or cultured cells were spun down, removed media, and suspended with corresponding antibody-containing 2% FBS/PBS. Cells were incubated for 30 min at room temperature in the dark and then washed once with 2%FBS/PBS.
Instrument	All cell populations were analyzed using a ThermoFisher Attune Nxt cytometer equipped with four lasers. GFP was detected using a 488 nm blue laser and a 510/10 bandpass emission filter; a 530/30 bandpass filter was used for experiments that converted to MEFL units. mTagBFP was detected using a 405 nm violet laser and 440/50 bandpass emission filter. iRFP720 was detected using a 638 nm red laser and 720/30 bandpass emission filter.
Software	All cytometry data was processed with FlowJo 10
Cell population abundance	We did not sort that cells.
Gating strategy	For FlowJo: A polygonal FSC-A/SSC-A gate was used to remove debris from the mammalian cell populations. Cell trace dye or fluorescence protein tag was used to differentiate immune cells from tumor cells.

- [x] Tick this box to confirm that a figure exemplifying the gating strategy is provided in the Supplementary Information.
